# Licoricidin triggers reactive oxygen species-mediated PANoptosis in human hepatocellular carcinoma cells

**DOI:** 10.7150/ijms.132175

**Published:** 2026-07-13

**Authors:** Ming-Chun Hung, Hui-Ling Chiou, Yi-Hsien Hsieh, Pei-Ni Chen, Yung-Luen Yu, Hsiang-Lin Lee

**Affiliations:** 1 Institute of Medicine, Chung Shan Medical University, Taichung, Taiwan.; 2 School of Medical Laboratory and Biotechnology, Chung Shan Medical University, Taichung, Taiwan.; 3 Department of Clinical Laboratory, Chung Shan Medical University Hospital, Taichung, Taiwan.; 4 Department of Medical Research, Chung Shan Medical University Hospital, Taichung, Taiwan.; 5 Institute of Translational Medicine and New Drug Development, China Medical University, Taichung, Taiwan.; 6 Center for Molecular Medicine, China Medical University Hospital, Taichung, Taiwan.; 7 Cancer Biology and Precision Therapeutics Center, China Medical University, Taichung, Taiwan.; 8 Office of Research and Development, Asia University, Taichung, Taiwan.; 9 School of Medicine, Chung Shan Medical University, Taichung, Taiwan.; 10 Deptartment of Surgery, Chung Shan Medical University Hospital, Taichung, Taiwan.

**Keywords:** hepatocellular carcinoma, Licoricidin, PANoptosis, ROS

## Abstract

Licoricidin (LCD), a natural isoflavonoid compound extracted from Glycyrrhiza species, has been extensively demonstrated to possess diverse biological activities, including anti-inflammatory and potent anti-cancer effects. However, the precise mechanism underlying LCD action against hepatocellular carcinoma (HCC) remains unclear, particularly regarding its regulation of cell death. In this study, we comprehensively explored the effects of LCD on HCC cells *in vitro* and investigated its role and mechanism of action in the induction of PANoptosis. Our results reveal that LCD exhibited potent anti-HCC activities by decreasing cell viability and significantly inhibiting clonogenic survival in HCC cell lines. Our results demonstrate that LCD triggered a substantial accumulation of reactive oxygen species and induced depolarization of the mitochondrial membrane, leading to profound mitochondrial dysfunction. We further confirmed that LCD activated a comprehensive PANoptosis program by synchronously upregulating the expression of apoptotic proteins (Bax, c-CASP3, and c-PARP1), pyroptotic proteins (c-CASP 1 and c-GSDMD), and the phosphorylation of necroptotic executioners (p-MLKL and p-RIPK1). Treatment with the ROS inhibitor (NAC), apoptosis inhibitor (ZVAD), or necroptosis inhibitor (Nec-1) significantly reduced the expression of PANoptosis-related proteins in LCD-treated cells. Furthermore, molecular docking simulations and cellular thermal shift assay (CETSA) assay confirmed the direct and stable binding of LCD to PANoptosis-related proteins. In summary, we show for the first time that LCD exerts favorable anti-HCC activities via the induction of PANoptosis through a ROS-dependent mechanism and potntial direct modulation of core executive proteins. This multi-target action suggests that LCD could be a novel candidate for the management of hepatocellular carcinoma.

## Introduction

Hepatocellular carcinoma (HCC) is the most common primary liver malignancy and a leading cause of cancer-related mortality worldwide 1. Despite curative options such as resection, ablation, and transplantation for early-stage disease, most patients present at advanced stages requiring systemic therapy 2,3. Current treatments, including sorafenib 4, regorafenib 5 and immune checkpoint inhibitors 6, provide limited survival benefits and are constrained by resistance and toxicity 7. Therefore, an urgent need for more effective therapeutic strategies against HCC.

Licoricidin (LCD), an isoflavan derived from *Glycyrrhiza* species (licorice), has attracted considerable pharmacological attention in recent years 8. Extensive pharmacological evidence indicates that LCD has been reported to possesses a broad spectrum of biological activities, including anti-inflammatory 9, antibacterial 10, antioxidant 11, and immunomodulatory effects 12. LCD exhibits robust antitumor efficacy in various tumor cells, such as suppressing cell proliferation 13, inducing cell cycle arrest and promoting apoptosis 14. Mechanistically, these effects are mediated through the disruption of key oncogenic axes such as MAPK 15 and AKT/NF-κB 16, as well as the inhibition of invasion and metastasis 17. Additionally, LCD has been shown to induce immunogenic cell death via endoplasmic reticulum stress 18.

Recent advances have broadened our understanding of cell death beyond canonical apoptosis to encompass other regulated and often immunogenic modalities, such as pyroptosis and necroptosis 19. PANoptosis has emerged as an integrative form of RCD characterized by the simultaneous activation of apoptosis, pyroptosis, and necroptosis 20, 21. This process is mediated by a multiprotein complex known as the PANoptosome and is thought to exert increased antitumor effects by circumventing cancer cell resistance to individual death pathways 22. PANoptosis has been implicated in a various tumors type. Previous studies have shown that PANoptotic activity is elevated in colorectal 23, lung 24, breast 25 and large B-cell lymphoma 26, as well as in hepatocellular carcinoma (HCC) 27. We hypothesized that the anti-tumor effects of LCD in HCC and the underlying molecular mechanisms of PANoptosome-related molecules remain to be elucidated.

## Materials and Methods

### Cell line and culture condition

Human HCC cell lines SK-HEP-1, Hep3B, Huh7, PLC/PRF/5, HA22T/VGH, and HepG2, and the normal hepatic stellate cell line TWNT-1, were obtained from the American Type Culture Collection (ATCC). SK-HEP-1 and TWNT-1 cells were cultured in Dulbecco's Modified Eagle Medium (DMEM, high glucose; Cytiva, #SH30003.02) supplemented with 10% fetal bovine serum (FBS; Cytiva HyClone™ Characterized Fetal Bovine Serum, #SH30396.03) and 1% penicillin-streptomycin (P/S; Corning, #30-002-Cl). HepG2 and HA22T/VGH cells were maintained in the same medium supplemented with 10% FBS, 1% P/S, 1% (v/v) non-essential amino acids (NEAA; Corning, #25-025-Cl), and 1% (v/v) sodium pyruvate (Corning, #25-000-Cl). Huh7 cells were cultured in DMEM (low glucose; Gibco, #31600-034) supplemented with 10% FBS, 1% P/S, and 1% (v/v) sodium pyruvate. Hep3B and PLC/PRF/5 cells were grown in Minimum Essential Medium (MEM; Gibco, #41500-067) supplemented with 10% FBS and 1% P/S. All cells were maintained at 37°C in a humidified incubator with 5% CO₂.

### Cell viability assay

Cell viability was evaluated using the MTT assay. SK-Hep-1, HepG2, Hep3B, PLC/PRF/5, Huh7, HA22T/VGH, and the normal hepatic cell line TWNT-1 were seeded into 96-well plates at a density of 2.5 × 10³ cells/well in 90 μL of complete medium and allowed to adhere overnight. The following day, cells were treated with various concentrations of LCD for 24 or 48 hours. After treatment, 10 μL of MTT solution (5 mg/mL) was added to each well and incubated for 2 hours at 37°C. The resulting formazan crystals were dissolved in 100 μL of isopropanol, and the absorbance was measured at 570 nm using a microplate reader. The half-maximal inhibitory concentration (IC₅₀) values were determined by nonlinear regression analysis using a dose-response inhibition (variable slope) model in GraphPad Prism 8 (GraphPad Software, San Diego, CA, USA).

### Colony formation assay

Hep3B, PLC/PRF/5, and Huh7 cells were seeded into 6-well plates at a density of 5 × 10² cells/well in 2 mL of complete medium and allowed to adhere overnight. The following day, cells were treated with various concentrations of LCD (0, 5 and 10 μM) and maintained for 7 days, with medium replacement every 2 days. At the end of the incubation period, colonies were fixed with methanol for 30 minutes and stained with 5% Giemsa solution for 4 hours at room temperature. Visible colonies were photographed and counted to evaluate the effects of LCD on clonogenic survival.

### Western Blot Analysis

Cells were lysed in NETN buffer (20 mM Tris-HCl, pH 8.0; 100 mM NaCl; 0.5 mM EDTA; 0.5% NP-40) supplemented with a protease inhibitor cocktail (Roche Diagnostics, Mannheim, Germany). Protein concentrations were determined using the Bradford protein assay (Bio-Rad, Hercules, CA, USA). Equal amounts of protein (10-20 μg) were separated by sodium dodecyl sulfate-polyacrylamide gel electrophoresis (8-13%) and transferred onto polyvinylidene difluoride membranes (PALL Corporation, Fort Washington, NY, USA). Membranes were blocked with 5% (w/v) skim milk in Tris-buffered saline containing 0.1% Tween-20 (TBST) for 1 h at room temperature, followed by incubation overnight at 4°C with primary antibodies diluted in TBST. After washing, membranes were incubated with horseradish peroxidase (HRP)-conjugated secondary antibodies (1:10000 dilution) for 1 h at room temperature. Finally, membranes were developed using enhanced chemiluminescence reagents (Millipore, Burlington, MA, USA) and detected using a chemiluminescence imaging system (Amersham ImageQuant 800, Amersham/GE, Buckinghamshire, UK). Band intensities were quantified using ImageJ software and normalized to GAPDH or β-Actin as loading controls. The following primary antibodies were used: anti-Bax (Santa Cruz Biotechnology; sc-7480; 1:1000), anti-cytochrome c (Santa Cruz Biotechnology, sc-13156; 1:1000), anti-c-CASP-1 (Asp297) (Affinity Biosciences, AF4005; 1:1000), anti-c-CASP3 (iREAL, IR96-401; 1:1000), anti-GSDMD (Santa Cruz Biotechnology, sc-393581; 1:1000), anti-MLKL (Abcam, ab183770; 1:1000), anti-p-MLKL (ABclonal, AP1174; 1:1000), anti-RIPK1 (Cell Signaling Technology, #3493; 1:1000), anti-p-RIPK1 S166 (Cell Signaling Technology, #65746; 1:1000), anti-PARP1 (Cell Signaling Technology, #9542; 1:1000), anti-GAPDH (Proteintech; 60004-1; 1:10,000) and anti-β-Actin (Santa Cruz Biotechnology, sc-47778; 1:10,000).

### Detection of Intracellular Reactive Oxygen Species (ROS)

The intracellular ROS levels of HCC cells were estimated using 2',7'-dichlorodihydrofluorescein diacetate (Invitrogen, Carlsbad, CA, USA). After the indicated treatments, cells were incubated with 10 μM H_2_DCFDA and 10 μM Hoechst 33342 at 37°C for 30 minutes in the dark. After incubation, the cells were gently washed with PBS. The fluorescence intensity of ROS production was visualized and captured using an ImageXpress Pico Automated Cell Imaging System (Molecular Devices, San Jose, CA, USA). Quantitative analysis of the fluorescent signal was performed using ImageXpress software (Molecular Devices), and the mean fluorescence intensity was calculated to compare relative ROS levels between different treatment groups.

### Apoptosis assay

Cell apoptosis analysis was evaluated using the Muse® Annexin V & Dead Cell Assay Kit (CYTEK®, MCH100105) according to the manufacturer's instructions. Hep3B and Huh7 cells were treated various concentrations of LCD (0, 5 and 10 μM) for 24 hours. After treatment, cells were harvested and stained with Muse™ Annexin V & Dead Cell Reagent for 10 min. The apoptotic status of cells was analyzed using the Guava Muse® Cell Analyzer (Millipre).

### Mitochondrial membrane potential assay

Cell apoptosis was analyzed using the Muse Mitopotential kit (Luminex, Austin, TX, USA). Hep3B and Huh7 cells were incubated with LCD at different concentrations (0, 5 and 10 μM) for 24 hours. Cells were collected and stained using the Muse Mitopotential kit for 30 min at 37°C. The mitochondria status of cells was monitored using the Guava Muse Cell Analyzer flow cytometer.

### Molecular docking

The three-dimensional (3D) crystal structures of human PARP1 (PDB ID: 7AAC), GSDMD (PDB ID: 7Z1X), MLKL (PDB ID: 4MWI) and RIPK1 (PDB ID: 4ITJ) were sourced from the RCSB Protein Data Bank (https://www.rcsb.org/). Proteins were prepared using the BIOVIA Discovery Studio 2025 Client. Subsequently, polar hydrogen atoms were added using PyRx (version 0.8) to optimize the potential for hydrogen bond interactions during docking. The chemical structure of LCD (PubChem CID: 480865) was retrieved from the PubChem database in SDF format. To ensure the most stable conformation for docking, the ligand underwent energy minimization using the Universal Force Field via the Open Babel engine. Molecular docking was performed using the Vina wizard integrated within PyRx. To ensure a comprehensive search for potential binding sites, we used a blind docking strategy and defined the grid box to cover the entire protein surface. The optimal pose with the lowest binding energy (highest affinity) was selected for further investigation. Protein-ligand intermolecular interactions, including hydrogen bonding and hydrophobic contacts, were visualized and analyzed using Discovery Studio 2025 Client.

### Cellular Thermal Shift Assay (CETSA)

The interaction between LCD and its potential target proteins (PARP1, RIPK, MLKL, and GSDMD), a cellular thermal shift assay (CETSA) was performed using Hep3B cell lysates. Cells were cultured and harvested by centrifugation, and resuspended in NETN lysis buffer containing 2 mM PMSF and a protease inhibitor cocktail. After three cycles of sonication (1 s on/off), lysates were centrifuged at 12,000 rpm for 20 min at 4°C to obtain soluble fractions. Supernatants were divided into two groups and treated with either 10 µM LCD or DMSO for 1 hours at room temperature. Samples were aliquot (60 µL) and heated at 43-58°C for 3 minutes, followed by cooling on ice and centrifugation to remove precipitated proteins. The remaining soluble proteins were analyzed by Western blotting to assess thermal stability and infer LCD targeting protein interactions.

### Statistical analysis

Each experiment was performed in triplicate, and the data are presented as the mean ± standard deviation (SD). Statistical analyses were performed using GraphPad Prism 8 (GraphPad Software, San Diego, CA, USA). Differences among multiple groups were assessed by one-way analysis of variance (ANOVA), followed by Tukey's post hoc test for multiple comparisons. * *p* < 0.05 and ** *p* < 0.01 compared with the control (0 μM) cells; # p < 0.05 compared with LCD treatment cells. N.S (no significant difference) compared with the control (0 μM) cells.

## Results

### LCD inhibits cell viability and colony formation in HCC cells

**Figure 1:**
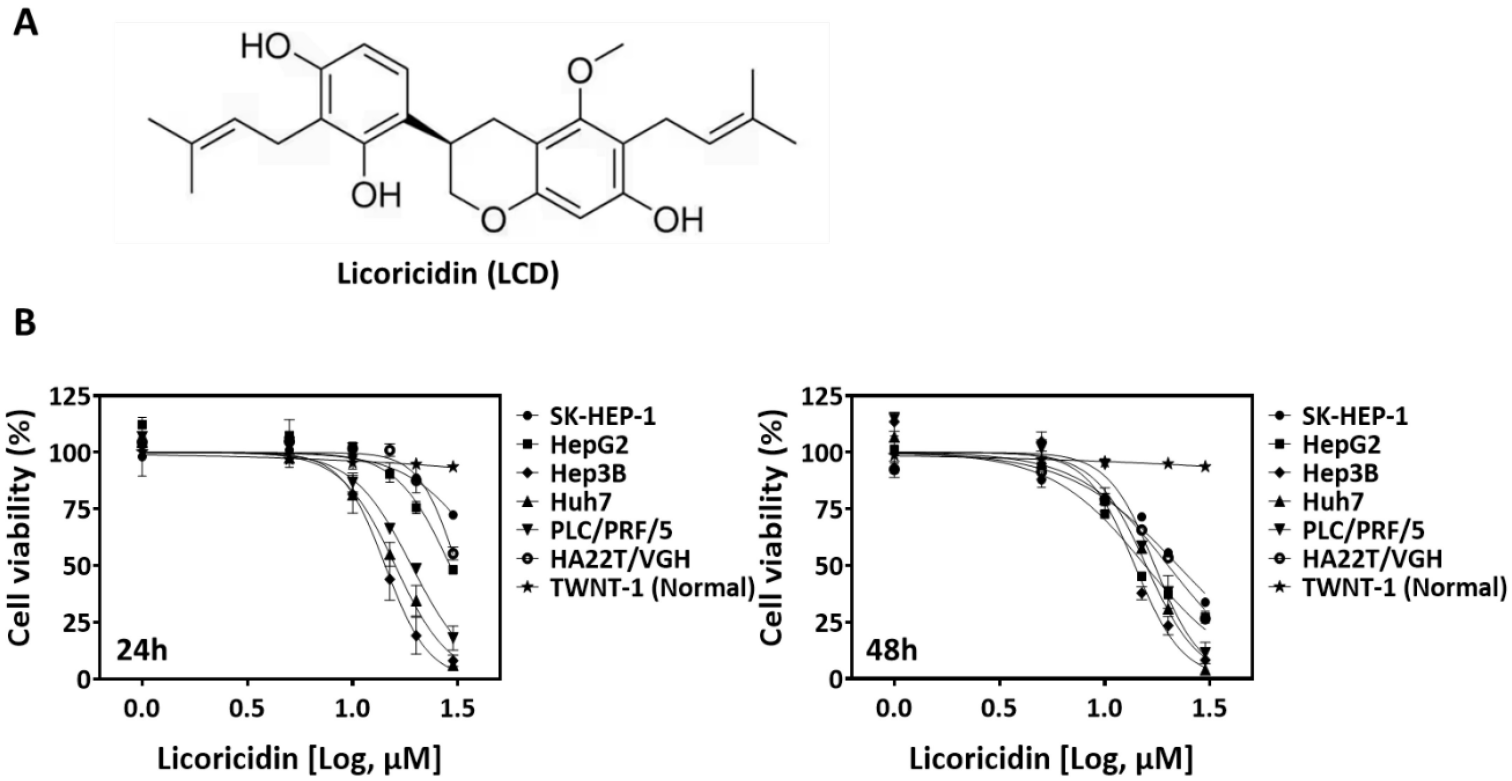
** LCD inhibits growth of HCC cells. (A)** Two-dimensional chemical structure of LCD. **(B)** The half-maximal inhibitory concentration (IC_50_) of LCD was evaluated in human HCC cell lines (HepG2, SK-HEP-1, HA22T/VGH, PLC/PRF/5, Hep3B, Huh7) and one immortalized normal human hepatic cell line (TWNT-1). Cells were treated with various concentrations (1, 5, 10, 15, 20, 30 μM) of LCD for 24 or 48 hours. Data are expressed as the mean ± SD of three independent experiments.

**Figure 2:**
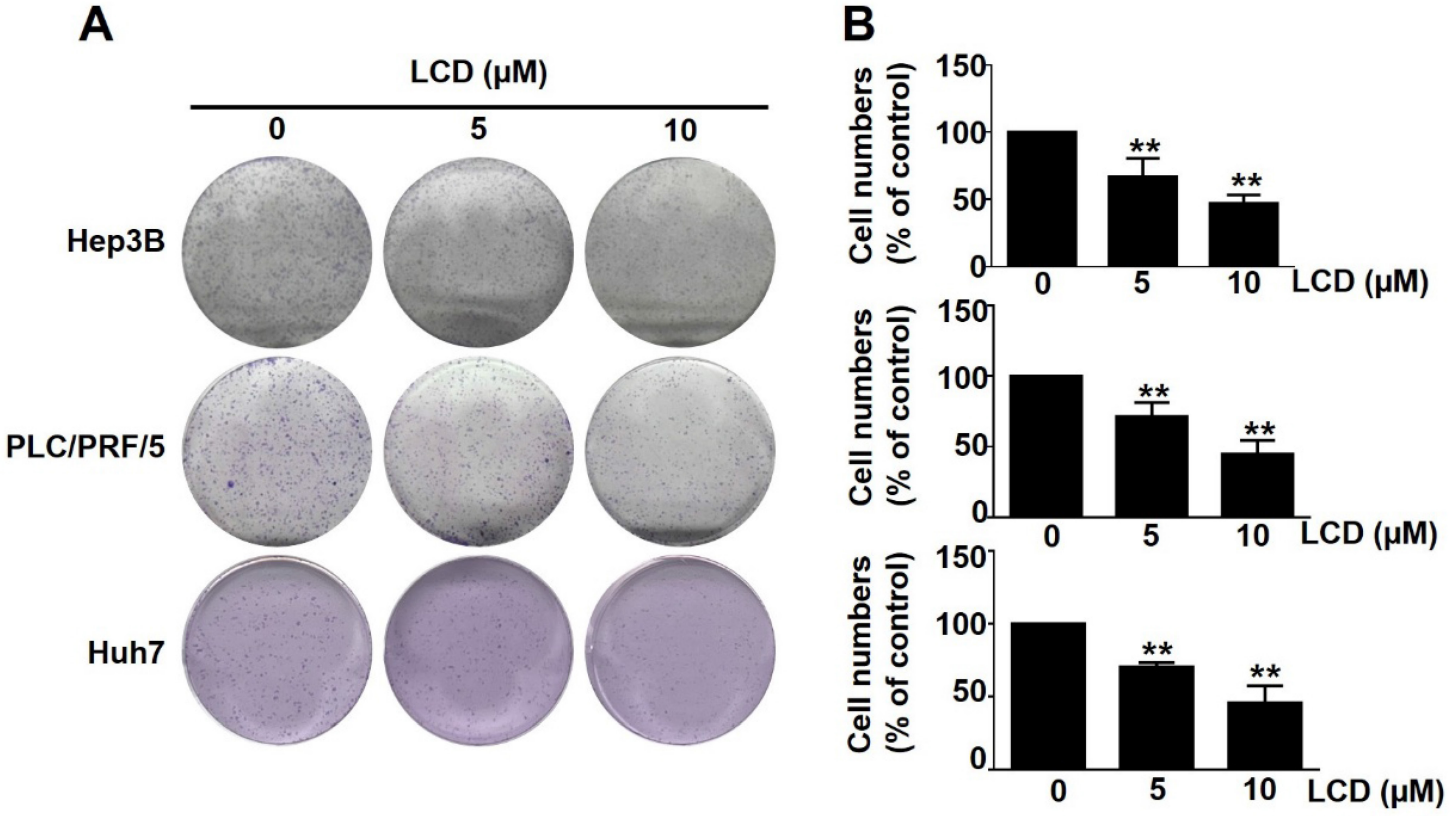
** LCD inhibits the proliferative rate of HCC cells. (A)** Representative images of the colony formation assay. Hep3B, PLC/PRF/5, and Huh7 cells were treated with indicated concentrations (0, 5, and 10 μM) of LCD and cultured for 7 days to allow tumor proliferation activity. **(B)** Quantitative analysis of the colony-forming number (% of control). Data are expressed as the mean ± SD of three independent experiments. ** *p* < 0.01 compared with the control (0 μM) cells.

The chemical structure of LCD is shown in Figure 1A. To investigate the anti-tumor potential of LCD in HCC, we first examined its effects on the viability of six human HCC cell lines (HepG2, SK-HEP-1, HA22T/VGH, PLC/PRF/5, Hep3B, and Huh7) and one normal hepatocyte cell line (TWNT-1). MTT assay results show that the viability of all HCC cell lines decreased in a dose-dependent manner after 24 and 48 hours of LCD treatment (Fig. 1B). Notably, the calculated IC50 values show that Hep3B, PLC/PRF/5, and Huh7 are the most susceptible cell lines. In contrast, TWNT-1 cells exhibited a significantly higher tolerance to LCD, suggesting that LCD exerts a selective cytotoxic effect on malignant cells. Furthermore, colony formation assays showed that LCD (5 and 10 μM) significantly inhibited the long-term proliferation of Hep3B, PLC/PRF/5, and Huh7 cells (Figure 2A, 2B), with cell number of colonies significantly reduced compared to the untreated control. These results suggested that LCD inhibit HCC cell viability and proliferation.

### LCD induced cell death and mitochondrial dysfunction in HCC cells

**Figure 3:**
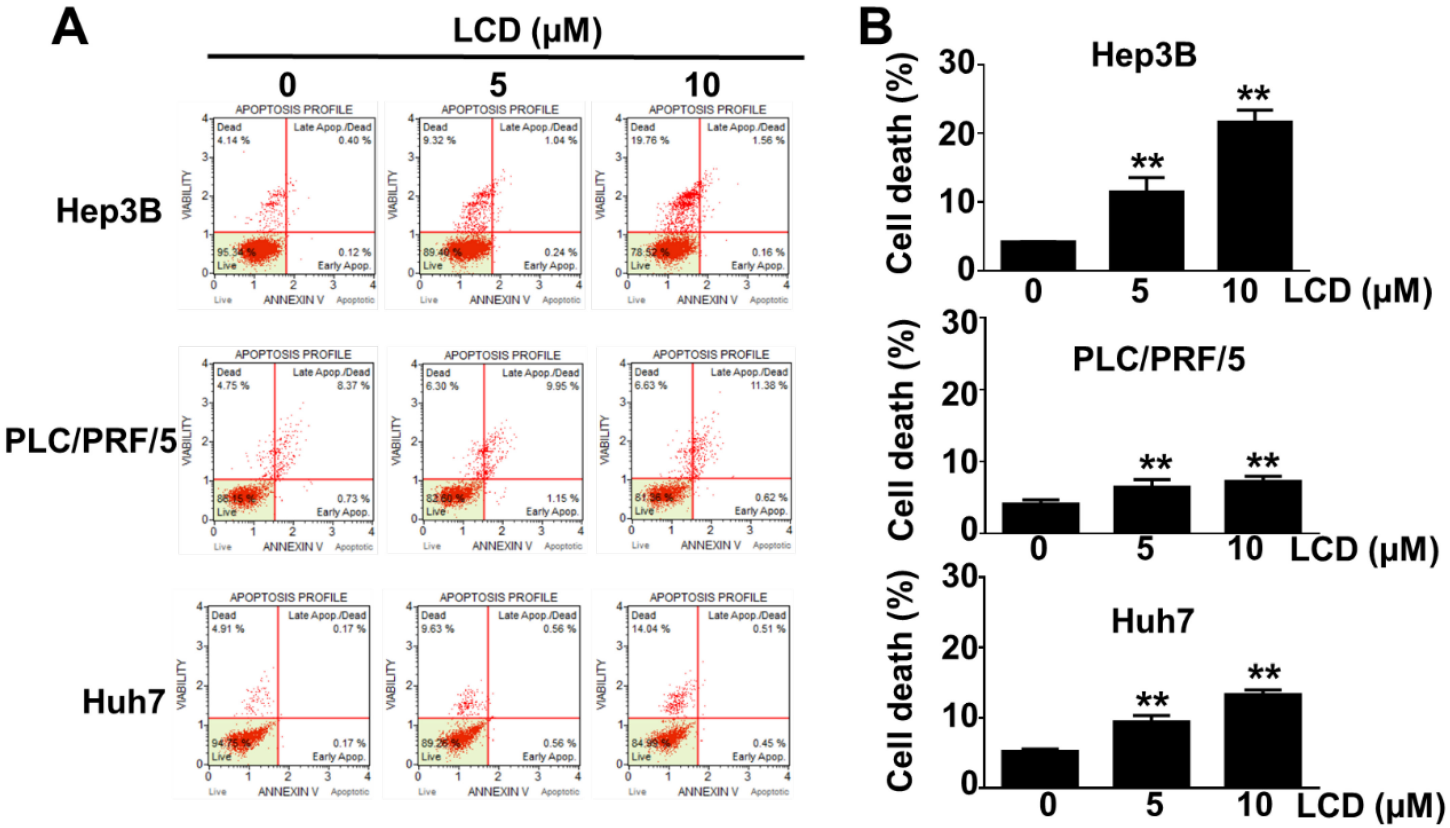
** LCD induces cell death in HCC cells. (A)** Flow cytometry analysis of cell death using Annexin V-FITC/PI dual staining. Hep3B, PLC/PRF/5, and Huh7 cells were treated with the indicated concentrations (0, 5, and 10 μM) of LCD for 24 hours. The quadrants represent live (Annexin V-/PI-), early apoptotic (Annexin V+/PI-), and late apoptotic/dead (Annexin V+/PI+) cells. **(B)** Quantitative analysis of the percentage of apoptotic/dead (Annexin V+/PI+) cells. Results are presented as the mean ± SD of three independent experiments. ** *p* < 0.01 compared with the control (0 μM) cells.

**Figure 4:**
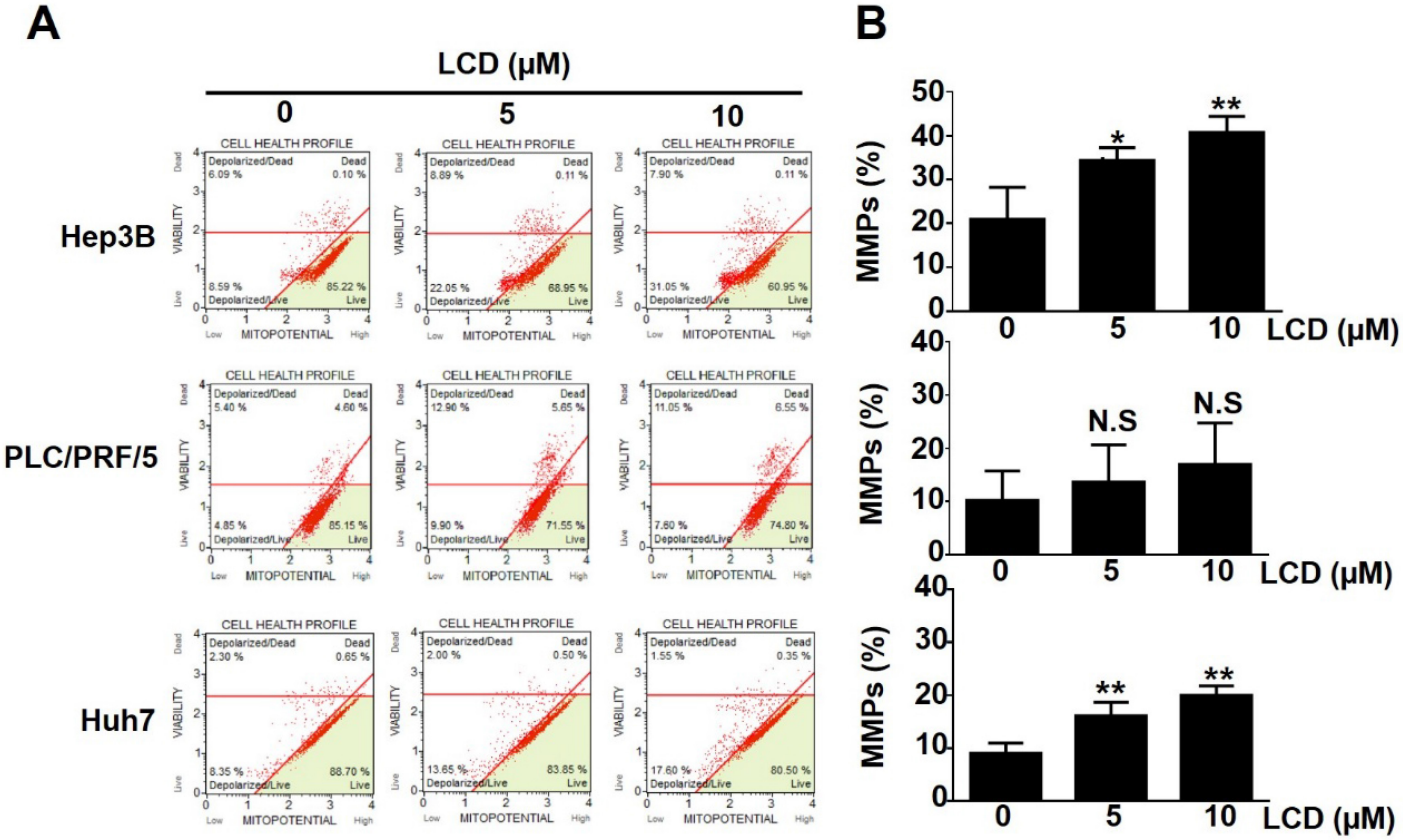
** LCD induces mitochondrial membrane potential depolarization in HCC cells. (A)** Representative flow cytometry plots of mitochondrial membrane potential (MMP) analyzed by JC-1 assay. Hep3B, PLC/PRF/5, and Huh7 cells were treated with the indicated concentrations (0, 5, and 10 μM) of LCD for 24 hours. **(B)** Quantitative analysis of the percentage of cells with depolarized mitochondria. Data are expressed as the mean ± SD of three independent experiments. * *p* < 0.05 and ** *p* < 0.01 compared with the control (0 μM) cells; N.S (no significant difference) compared with the control (0 μM) cells.

To further characterize the LCD-induced growth inhibition mechanism, we performed Annexin V-FITC/PI dual staining using flow cytometry. As shown in Figure 3A, 3B, LCD treatment significantly increased the proportion of dead Hep3B, PLC/PRF/5, and Huh7 cells, and the proportion of Annexin V-positive cells increased in a dose-dependent manner. Given that mitochondrial dysfunction is a marker of multiple cell death pathways, we used the mitochondrial membrane potential probe JC-1 to assess the mitochondrial membrane potential (MMP). LCD exposure triggered significant MMP depolarization, particularly in Hep3B and Huh7 cells (Figure 4A, 4B), suggesting that LCD-induced cell death is associated with mitochondrial dysfunction.

### LCD induced PANoptosis in HCC cells

**Figure 5:**
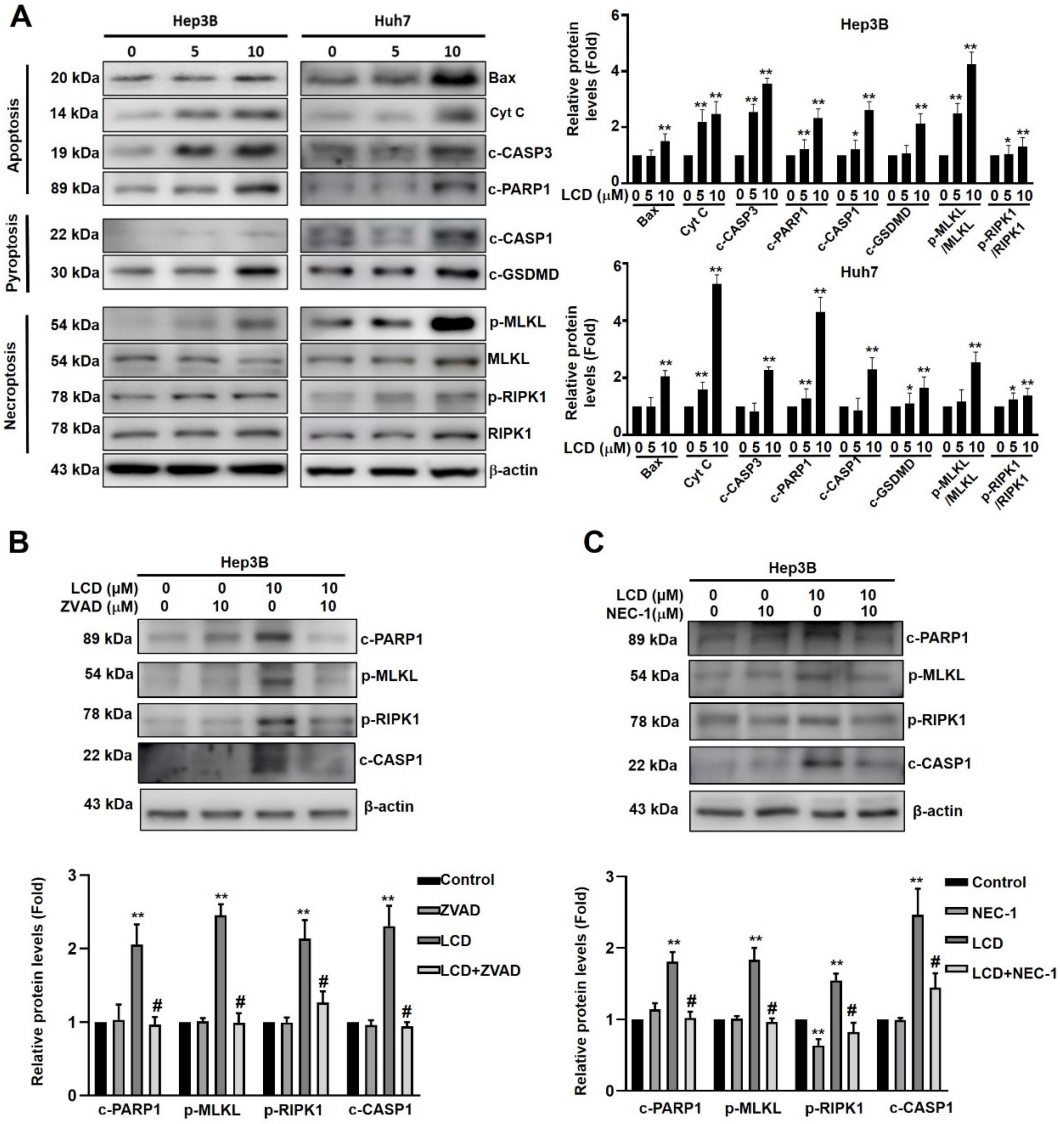
** LCD triggers PANoptosis in HCC cells. (A)** Protein expression levels of Bax, cytochrome c and key markers for PANoptosis were assessed by Western blot analysis in Hep3B and Huh7 cells treated with the indicated concentrations (0, 5, and 10 μM) of LCD for 24 hours. β-actin was used as an internal loading control. **(B)** Hep3B cells were pretreated with the ZVAD (caspase inhibitor; 10 µM) or **(C)** Nec-1 (necroptosis inhibitor;10 µM) for 2 hours, followed by LCD treatment for 24 hours. The expression levels of PANoptosis-related proteins (c-PARP1, p-RIPK1, p-MLKL, and c-CASP1) were detected by Western blot analysis. Data is presented as the mean ± SD of three independent experiments. * *p* < 0.05 and ** *p* < 0.01 compared with the control (0 μM) cells, # *p < 0.05* compared with LCD treatment cells.

To determine whether cell death ultimately leads to PANoptosis, we analyzed the expression of key markers for apoptosis, pyroptosis, and necroptosis using western blot analysis. As shown in Figure 5A, LCD treatment upregulated the markers of apoptosis (Bax, Cytochrome c, c-CASP3, and c-PARP1). Simultaneously, LCD activated the pyroptosis pathway, as evidenced by increased levels of c-caspase-1 (c-CASP1) and c-GSDMD. Furthermore, necroptosis markers, including the phosphorylation of MLKL (p-MLKL) and RIPK1 (p-RIPK1), were significantly elevated. To examine the PANoptosis pathway whether involved in LCD-treared HCC cell death, using caspase inhibitor (ZVAD) or necroptosis inhibitor (Nec-1) treatment significantly decreased the expression of these PANoptosis-associated proteins (c-PARP1, p-MLKL, p-RIPK1, c-CASP1) in LCD-treated Hep3B cells (Figure 5B, 5C). These findings collectively demonstrate that LCD effectively triggers PANoptosis in HCC cells.

### LCD induces ROS-mediated PANoptosis in HCC cells

**Figure 6:**
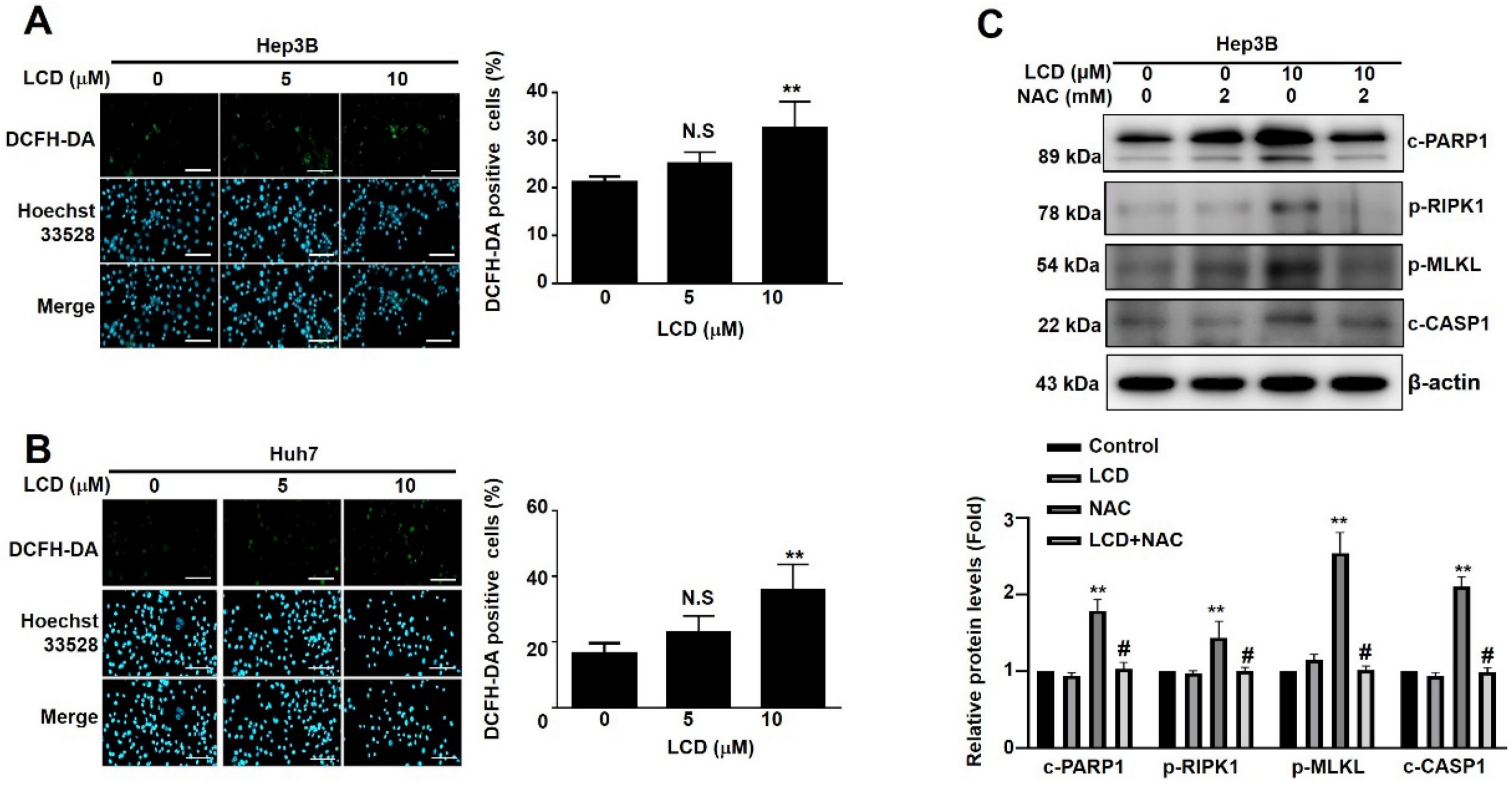
** LCD triggers PANoptosis by induction of intracellular ROS in human HCC cells. (A. B)** Representative fluorescence images of ROS levels detected by DCFH-DA staining. Hep3B and Huh7 cells were treated with the indicated concentrations (0, 5, and 10 μM) of LCD for 24 hours. The nuclei were counterstained with Hoechst 33342 (blue); green fluorescence indicates ROS production. Quantitative analysis of DCFH-DA positive cells (%). **(C)** Pretreated with the NAC (ROS inhibitor; 2 mM) for 2 hours, followed by LCD treatment for 24 hours. The expression levels of PANoptosis-related proteins (c-PARP1, p-RIPK1, p-MLKL, and c-CASP1) were detected by Western blot analysis Data are presented as the mean ± SD of three independent experiments. * *p* < 0.05 and ** *p* < 0.01 compared with the control (0 μM) cells; N.S (no significant difference) compared with the control (0 μM) cells. Scale bar = 20 µm.

Oxidative stress is often a key driver of programmed cell death. Using DCFH-DA staining, we observed that LCD treatment (10 μM) significantly increased the levels of ROS in both Hep3B and Huh7 cells (Figure 6A. 6B). To determine whether this oxidative damage and cell death ultimately leads to PANoptosis, our results showed that LCD (20 µM) markedly increased the expression of PANoptosis-related proteins, including c-PARP1, p-RIPK1, p-MLKL and c-CASP1. In contrast, ROS scavenger NAC (2 mM) treatment significantly attenuated the expression of these PANoptosis-associated proteins in LCD-treated Hep3B cells (Figure 6C). These findings collectively demonstrate that LCD effectively triggers ROS-mediate PANoptosis in HCC cells.

### Structural evidence for the direct interaction between LCD and key PANoptotic executive proteins

**Figure 7:**
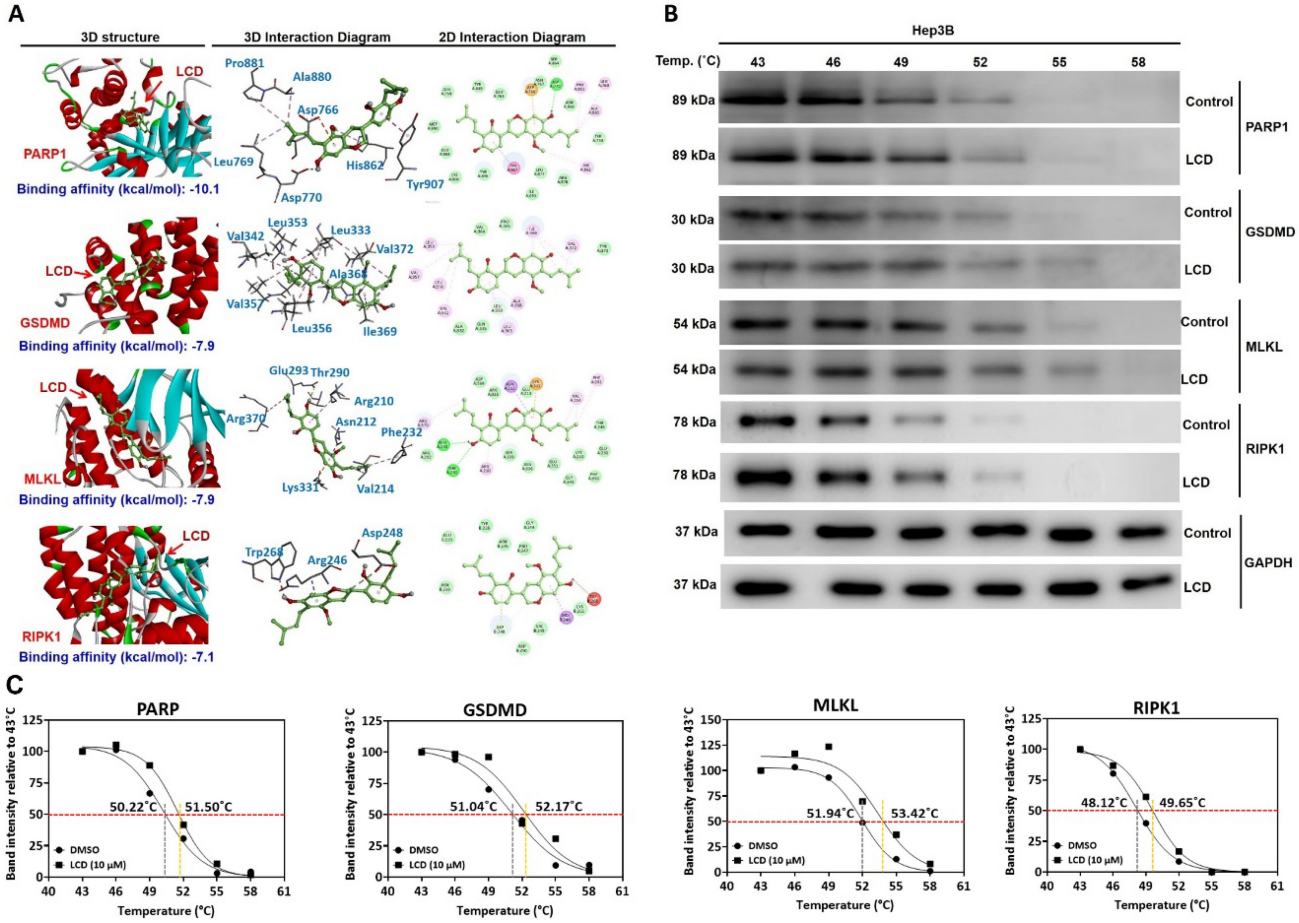
** Molecular docking analysis of LCD binding to key proteins involved in PANoptosis. (A)** Computational molecular docking analysis was performed to evaluate the binding potential of LCD to PARP1, GSDMD, MLKL, and RIPK1. The 3D structures, 3D interaction diagrams, and 2D interaction diagrams are displayed for each target. LCD exhibited high binding affinities toward PARP1 (-10.1 kcal/mol), GSDMD (-7.9 kcal/mol), MLKL (-7.9 kcal/mol), and RIPK1 (-7.1 kcal/mol). LCD may directly modulate the activity of these executive proteins, thereby triggering PANoptosis in HCC cells.** (B)** The CETSA was performed to assess changes in the thermal degradation rate of PARP1, GSDMD, MLKL and RIPK1 protein at increasing temperatures with or without LCD treatment and detected with western blotting. GAPDH as was used as an internal loading control. **(C).** Thermal melting curves for PARP1, GSDMD, MLKL and RIPK1. Data are presented as the mean ± SD of three independent experiments.

To explore the potential direct interaction between LCD and the PANoptosis mechanism, we performed computational molecular docking analysis (Figure 7A). Consistent with our western blot findings, LCD exhibited high binding affinity for the executive proteins in all three pathways. The predicted binding energies for LCD with PARP1, GSDMD, MLKL, and RIPK1 were -10.1 kcal/mol, -7.9 kcal/mol, -7.9 kcal/mol, and -7.1 kcal/mol, respectively (Figure 7A). The docking models identified key amino acid residues involved in hydrogen bonding and hydrophobic interactions, providing structural evidence for the ability of LCD to directly modulate these executioners and initiate the PANoptosis process.

To assess the direct binding between LCD and its target proteins, a cellular thermal shift assay (CETSA) was performed using Hep3B cell lysates. Our results found that LCD (10 μM) significantly increased the thermal stability of PARP1, RIPK1, GSDMD, and MLKL compared to the vehicle control (Figure 7B). Thermal melting curves revealed a clear rightward shift following LCD treatment. Specifically, the melting temperature (Tm) increased from 50.22°C to 51.50°C for PARP, 51.04°C to 52.17°C for GSDMD, 51.94°C to 53.42°C for MLKL and 48.12°C to 49.65°C for RIPK1 (Figure 7C). These results support that LCD directly interacts with PANoptotict-related proteins, contributing to modulation of the PANoptotic pathway.

## Discussion

**Figure 8:**
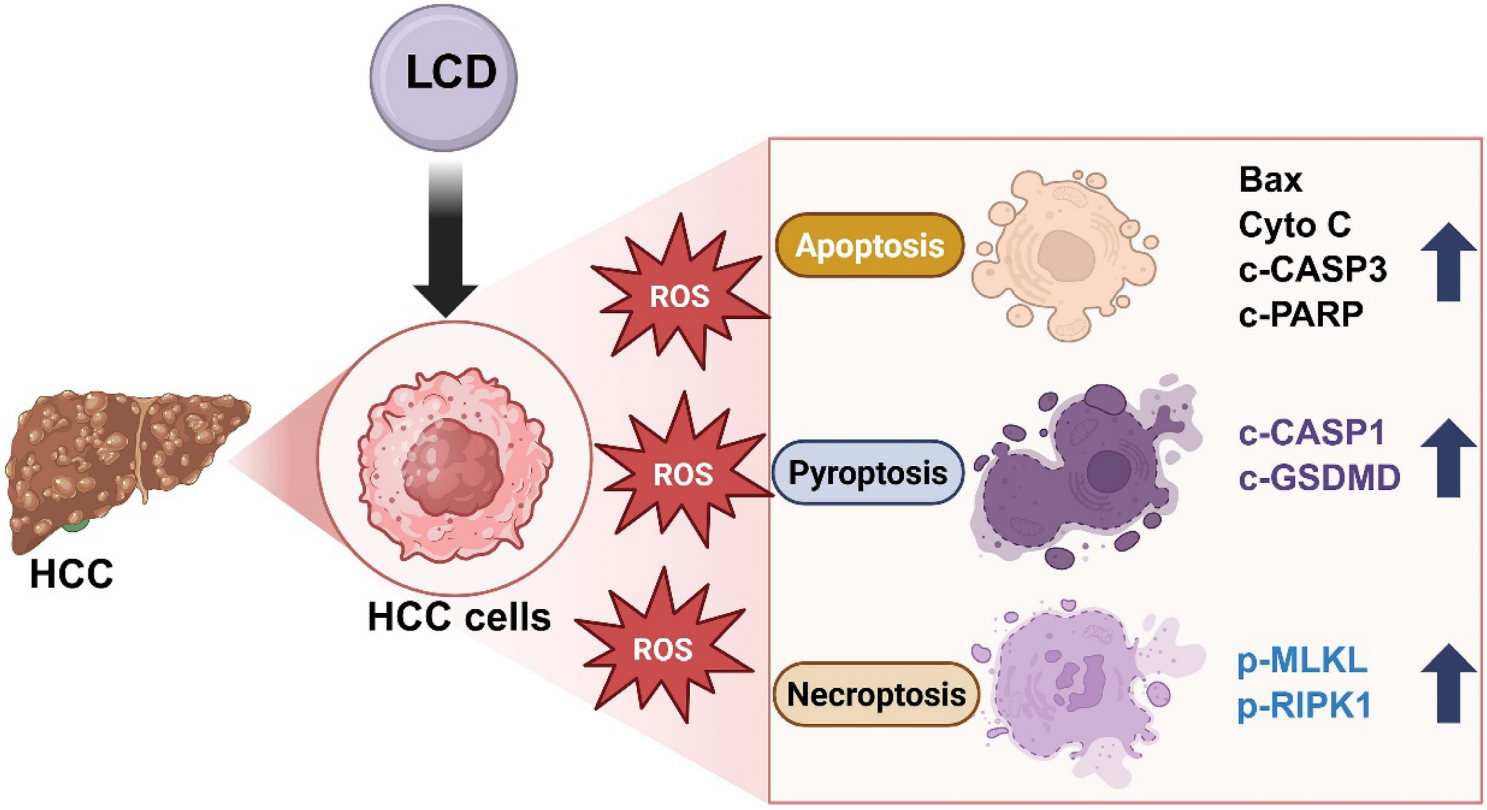
** LCD induces ROS-mediated PANoptosis in HCC cells.** Schematic illustration that LCD elevates intracellular ROS, causing mitochondrial dysfunction and activating apoptosis (Bax, Cyto c, c-CASP3, c-PARP1), pyroptosis (c-CASP1, GSDMD), and necroptosis (p-MLKL, p-RIPK1). These findings indicate that LCD triggers ROS targeting PANoptosis in hepatocellular carcinoma cells.

Emerging evidence indicates that PANoptosis is an integrated programmed cell death (PCD) pathway that coordinates apoptosis, pyroptosis, and necroptosis. By simultaneously activating multiple lethal pathways, PANoptosis provides a powerful mechanism to circumvent the resistance issues associated with single-pathway failure 28, 29. In this study, we demonstrated that LCD significantly inhibits HCC cell viability and synchronously activates apoptosis (Bax, Cytochrome c, c-CASP3, c-PARP1), pyroptosis (c-CASP1, c-GSDMD), and necroptosis (p-MLKL, p-RIPK1), thereby inducing multiple cell death characteristics of PANoptosis (Figure 8).

Recent years, PANoptosis has emerged as a multifaceted form of regulated cell death that integrates diverse molecular targets and signaling pathways, representing a promising therapeutic strategy for cancer treatment 30; however, effective pharmacological agents targeting this process remain limited. Emerging evidence suggests that various anticancer drugs or natural compounds exert antitumor effects by inducing PANoptosis in different tumor types 31. For example, ABT-737 targets anti-apoptotic Bcl-2 family proteins to trigger PANoptosis, leading to significant inhibition of HCC cell growth, proliferation, migration, invasion, and *in vivo* tumor progression 32. In addition, Li et al. reported that Atramacronoid A (AM-A) induces PANoptosis and mitochondrial dysfunction in breast cancer cells through coordinated activation of the CASP3/PARP, GSDMD, and MLKL pathways 33. Dr Gu et al., suggested that Tetrahydromagnolol (THM) targeting TRIM38-dependent PANoptosis and exhibits synergistic antitumor effects in combination with standard anticancer reagents (Cetuximab, FOLFOX, and FOLFIRI regimens) against human colorectal cancer cells 34. Furthermore, Gao et al. demonstrated that the combination of paclitaxel and cephalomannine induces PANoptosis in TNBC cells via ROS-mediated cell death 35. These findings are consistent with our results, demonstrating that LCD induces PANoptosis through ROS-mediated cell death pathways in HCC cells.

LCD exhibits potent inhibitory activity across six HCC cell lines, particularly in Hep3B and Huh7 cells. This selective inhibitory profile is consistent with previous reports on licorice-derived compounds, highlighting their potential to suppress malignant growth while sparing normal hepatic tissue 11, 18. We found that LCD significant increase apoptosis, reflecting a widespread loss of plasma membrane integrity. This phenomenon is consistent with the observed upregulation of c-GSDMD and p-MLKL. Since both pyroptosis and necroptosis ultimately lead to cell membrane rupture, these observations provide evidence that LCD simultaneously initiates multiple PCD pathways. Furthermore, the accumulation of ROS and mitochondrial dysfunction are considered critical upstream regulators of the PANoptotic cascade 20. Increased oxidative stress leads to the depolarization of mitochondrial membrane potential (MMP), and oxidative phosphorylation (OXPHOS) facilitating the release of pro-death factors 36. One report has been found that LCD induces cell apoptosis and suppresses metastasis by targeting ICMT/Ras pathway ingastric cancer 37. Our results demonstrate that LCD significantly increased intracellular ROS levels in Hep3B and Huh7 cells, accompanied by a significant decrease in MMPs. Simultaneously, increased oxidative stress is associated with activation of CASP-1, which cleaves GSDMD to generate its N-terminal fragment (cleaved GSDMD). This fragment translocates to the plasma membrane and forms pores, resulting in loss of membrane integrity, a hallmark of pyroptosis 38. In addition, ROS accumulation promotes RIPK1 phosphorylation, facilitating its interaction with RIPK3 to form the necrosome, which subsequently induces MLKL phosphorylation (p-MLKL) and leads to necroptotic cell lysis. RIPK1 serves as a scaffold for the assembly of the PANoptosome complex, while GSDMD and MLKL are key executive proteins for cell lysis 39. As shown in Figure 6C, our results demonstrated that NAC treatment significantly decreased the expression of PANoptosis-related proteins in LCD-treated Hep3B cells. These findings indicate that LCD triggers ROS-mediated PANoptosis in HCC cells. From molecular docking analysis, we found that LCD has a strong potential binding affinities for GSDMD (-7.9 kcal/mol), MLKL (-7.3 kcal/mol), and RIPK1 (-7.5 kcal/mol), with binding sites located within their functional domains. LCD enhances cleavage activity via binding to GSDMD and MLKL, while interacting with RIPK1 to promote PANoptosome assembly, confirming these proteins as direct targets mediating HCC cell death.

In this study has several limitations that should be addressed. In our findings are primarily based on *in vitro* cell line models, and the lack of *in vivo* animal model to validate the anti-tumor efficacy and PANoptosis-inducing effects of LCD, such as human HCC organoid models (HCC patient-derived organoids) or *in vivo* HCC xenograft models. In addition, using gene silencing approaches to delineate the precise contribution of individual PANoptotic pathways and further elucidate the mechanistic roles of PANoptosis regulators. These future directions will help to validate and extend our current findings and enhance the translational significance of LCD as a potential anti-HCC therapeutic agent.

## Conclusion

In this study, we demonstrated that the antitumor effects of LCD are mediated through ROS-induced mitochondrial dysfunction and potential direct interactions with PANoptosis-related proteins, thereby offering a strategic advantage in overcoming apoptosis resistance in HCC. These findings not only deepen our understanding of the pharmacological actions of LCD but also highlight the induction of PANoptosis as a promising therapeutic strategy for hepatocellular carcinoma.
